# Effects of Thifluzamide Treatment on the Production of Cell Wall Degrading Enzymes in *Rhizoctonia solani* and Phenylpropane Metabolism in Pear Fruit

**DOI:** 10.3390/pathogens13110963

**Published:** 2024-11-05

**Authors:** Yushuo Wu, Weiwei Yan, Xiaonan Sun, Xinnan Zhang, Yonghong Ge, Xiaohui Jia

**Affiliations:** 1 Institute of Pomology, Chinese Academy of Agricultural Sciences, Xingcheng 125100, China; wys18641842240@163.com (Y.W.); yanweiwei@caas.cn (W.Y.); xiaonansun_job@163.com (X.S.); zhangxinnan@caas.cn (X.Z.); 2 College of Food Science and Engineering, Bohai University, Jinzhou 121013, China

**Keywords:** *Rhizoctonia solani*, thifluzamide, pear fruit, cell wall-degrading enzyme, phenylpropanoid pathway

## Abstract

The study aimed to investigate the effects of thifluzamide (2.67 mg/L) on ‘Huangguan’ pear fruit rot caused by *Rhizoctonia solani* during storage, as well as the activities of polygalacturonase (PG), pectin methylesterase (PME), polygalacturonic acid trans-eliminase (PGTE), pectin methyl trans-eliminase (PMTE), xylanase, and pectate lyase (PL) secreted by *R. solani*. The results showed that thifluzamide treatment significantly inhibited the activities of PG, PME, PGTE, PMTE, xylanase, and PL secreted by *R. solani* after 3 days in vitro culture, compared to the control. Thifluzamide also increased the activities of phenylalanine ammonia-lyase (PAL), cinnamate-4-hydroxylase (C4H), and 4-coumarate CoA ligase (4CL), and the contents of flavonoids and total phenolic compounds in pear fruit. Furthermore, thifluzamide increased the expression of *PcPAL*, *PcC4H*, *Pc4CL*, *Pcβ-1,3-GA*, *PcLCH*, *PcF3H*, and *PcDFR* involved in phenylpropanoid metabolism in pear fruit. In conclusion, thifluzamide treatment reduced the infection ability of *R. solani* by inhibiting the expression of the genes encoding cell wall-degrading enzymes in *R. solani*. At the same time, it inhibited the activities of cell wall-degrading enzymes induced resistance against *R. solani* infection in ‘Huangguan’ pears by promoting phenylpropane metabolism.

## 1. Introduction

Pear (*Pyrus*) is a popular fruit belonging to the Rosaceae family, a diverse and economically vital group. Pears originated in Asia, and modern production is centered in the temperate regions of China, the U.S., the E.U., and Argentina [1]. While there are over 3000 varieties of pears, the most commonly available are European (*Pyrus communis*) and Asian pear (*Pyrus Pyrifolia*) [2]. Pears are an excellent source of nutrients, as they are rich in polyphenols, minerals, vitamins, amino acids, volatile oils, and triterpenes [3]. Pear is an important fruit crop worldwide. China’s pear production has more than tripled in the last two decades, from 5.5 million metric tons to 19.0 million metric tons, accounting for 70% of the world’s pear production [4]. ‘Huangguan’ pear is a hybrid between the pear varieties ‘Xuehua’ (*Pyrus bretschneideri* L. cv. Xuehua) and ‘New century’ (*Pyrus pyrifolia* Nakai cv. Shinseiki) [5]. This variety is cherished by both producers and consumers due to its many excellent characteristics, such as its beautiful appearance, strong stress resistance, and early fruiting [6] (Ma et al., 2016). However, a newly emerging postharvest fungal disease, known as *Rhizoctonia solani*, poses a potential threat to the storing process of ‘Huangguan’ pears [7].

*R. solani* is the largest and most complex multinucleated *Rhizoctonia* sp. The asexual state of *R. solani* belongs to the *Rhizoctonia* spp. The sexual state, *Thanatephorus curcuris* (Frank) Donk, belongs to the *Basidiomycetes*, *Thanatephorus cucumeris*. In general, most of them are asexual in the field, making it difficult to locate a specimen in its sexual phase [8] (Ning et al., 2019). Pears are not the sole victims of *R. solani*. For example, *R. solani* infects all subterranean parts of potato plants, including tubers, shoots, roots, stolons, and stems. Black dandruff usually occurs on the surface of infected potato tubers and forms sclerotia. This can cause stem canker and black scurf [9]. Rice sheath blight is similarly caused by *R. solani*. It causes a loss of approximately 50% of global rice yield [10]. *R. solani* exists in rice as sclerotia or hyphae, infecting rice plants by forming infection pads and/or foliar appressorium on the plant surface. After the initial infection, the pathogen moves up through the surface hyphae and forms new structures in rice, which is known as rice sheath blight [11].

The ability to penetrate the strong physical barrier of plant cell walls is facilitated by the secretion of cell wall-degrading enzymes (CWDEs) by various pathogens. CWDEs can degrade a wide range of complex, cross-linked polysaccharides and glycoproteins. In addition to infiltration of cells, CWDEs play a role in releasing nutrients for use by pathogens and are important determinants of pathogenicity. CWDEs are mainly divided into cellulase, pectinase, hemicellulose, and keratinase. Both cellulase and pectinase are the main pathogenic factors, playing an important role in the development of the disease [12]. The differences in pathogenicity among different strains are closely related to these enzymes. A higher activity of CWDEs may cause stronger pathogenicity of the strain [13].

The phenylpropanoid pathway is the main secondary metabolic pathway, playing an important role in disease resistance [14]. The three key enzymes in the phenylpropane metabolism include phenylalanine ammonia-lyase (PAL), cinnamic acid 4-hydroxylase (C4H), and 4-coumarate Coenzyme A ligase (4CL). This pathway can be used to convert phenylalanine for the synthesis of typical antibacterial substances, such as phenolic acids, flavonoids, and lignin [15]. Studies have shown that the disease resistance of sweet cherry, blueberry, and pear was improved by activating the phenylpropanoid pathway [16]. PAL, a rate-limiting enzyme in the phenolic synthesis pathway, catalyzes the conversion of phenylalanine to trans-cinnamic acid, a precursor for the synthesis of lignin, flavonoids, and coumarin [17,18]. C4H catalyzes the hydroxylation of cinnamic acid to coumaric acid, which is, in turn, catalyzed by a series of enzymes to produce caffeic, ferulic, and sinapic acids [19]. 4CL catalyzes the conversion of p-coumaric acid, ferulic acid, caffeic acid, and cinnamic acid to the counterpart, phenolic acid-CoA [20]. Regulation of the phenylpropane metabolism can promote the accumulation of phenolic substances and increase the resistance of fruit to pathogenic microorganisms [15].

The study aimed to investigate the role of thifluzamide, a broad-spectrum succinate dehydrogenase inhibitor fungicide, in regulating cell wall degradation by altering the activity of softening-related enzymes and regulating disease resistance in postharvest fruits. The effects of thifluzamide treatment on CWDEs secreted in vitro by *R. solani* and the activity and gene expression of key enzymes involved in phenylpropane metabolism in ‘Huangguan’ pear fruit were determined. 

## 2. Materials and Methods

### 2.1. Pathogen

*R. solani* was obtained from the Postharvest Disease Research Group, Fruit Research Institute, Chinese Academy of Agricultural Sciences, and cultured on Petri dishes in potato dextrose agar (PDA) (Solarbio Life Science, Shanghai, China) at 25 °C for 3 days. The Petri dishes with overgrown mycelia were then stored at 4 °C.

### 2.2. Fruit and Treatment

‘Huangguan’ pears with similar size, surface color, and absence of mechanical injuries were collected from a well-managed commercial orchard in Xinji, Hebei Province, China, shipped to the Postharvest Laboratory at the harvest day and divided into four batches (150 fruits per batch). For the protective trial, two batches of the selected fruit were immersed in distilled water (control), and the other batch was immersed in 2.67 mg L^−1^ thifluzamide (Yancheng Limin Chemical Co., Ltd., Yancheng, China) solution for 10 min. After drying, the pears were wounded by making punctures (5 mm in diameter and 2 mm in depth) with a sterilized borer. One group of fruits treated with distilled water was inoculated with PDA (5 mm × 5 mm), and another group of fruits treated with thifluzamide were inoculated with pathogen blocks (5 mm × 5 mm). For the therapeutic trial, two batches of the selected fruit were inoculated with pathogen blocks using the above method, followed by dipping in 2.67 mg L^−1^ thifluzamide solution and distilled water for 10 min. All the fruits were then stored in a humid and warm environment (25 °C and 90–95 % RH) to promote fungal growth. 

### 2.3. Sample Collection

Samples were collected using the method of Li et al. [21]. On the 0, 2, 4, 6, 8, and 10 days, pulp tissue was taken from 1–2 cm around the lesion spot in each treatment and stored at −80 °C for biochemical analysis.

### 2.4. Preparation of Crude Enzyme

Holes with a diameter of 5 mm were drilled on the edge of the pathogen colonies growing on PDA medium with a hole perforator, and five mycelium blocks were selected with an inoculation needle and transferred to 100 mL PDB medium. After shaking culture at 25 °C for 3 days, 0.267 mg of thifluzamide was added to one group of culture medium, while the other group was not added with any substance as a control, and the shaking culture was continued. Samples (5 mL of incubation culture) were taken at 0, 6, 12, 24, 48, 72 and 96 h. Samples were collected for each time. After centrifugation at 10,000 r/min and 4 °C for 30 min, the sediment was discarded, and the supernatant was crude enzyme solution.

### 2.5. Assay of the Activity of CWDE

The activities of PG and PME were assayed following the method by Jia et al. [22] with minor modifications. Briefly, the reaction mixture consisted of 1.0 mL acetic acid-sodium acetate buffer (50 mmol/L, pH 5.5) and 0.5 mL substrate (10 g/L polygalacturonic acid for PG substrate and 10 g/L pectin for PME substrate). The mixture was put into two test tubes, and 0.5 mL of enzyme extract was added to one test tube after incubation at 37 °C for 5 min. The other test tube was added with the same amount of inactivated enzyme solution (used as a control). The two tubes were kept at 37 °C for 1 h with full shaking, and 1.5 mL DNS was added immediately after the end of the insulation. The absorbance value at 540 nm was measured by placing the mixture in a beaker with boiling water for 5 min and then rapidly cooling it to room temperature with cold water. The activity (U·mL^−1^) was expressed as one enzyme activity unit (U) per minute per milliliter of enzyme solution, catalyzing the production of 1 g of galacturonic acid at 37 °C.

The activity of xylanase was measured following the method of Yuan et al. [23] with minor modifications. The reaction solution consisted of 1.0 mL acetic acid-sodium acetate buffer (50 mmol/L, pH 5.5) and 0.5 mL substrate (10 g/L xylan for xylanase). The mixture was put into two test tubes, and 0.5 mL of the enzyme extract was added to one test tube after incubation at 37 °C for 5 min. The other test tube was used as a control and added with the same amount of inactivated enzyme solution. After fully shaking and mixing, the mixture was kept at 37 °C for another 1 h, and then 1.5 mL 3,5-dinitrosalicylic acid (DNS) was added to the reaction mixture immediately. The absorbance value was measured at 540 nm after being boiled in a beaker with boiling water for 5 min and then quickly cooled to room temperature with cold water. The activity (U·mL^−1^) was expressed as one enzyme activity unit (U) per minute per milliliter of enzyme solution, catalyzing the production of 1 g of galacturonic acid at 37 °C.

The activity of pectic lyase was analyzed following Ahn et al.’s method [24] with minor modifications. Briefly, 1 mL of crude enzyme solution and 2 mL of 1% pectin solution were placed in two test tubes, respectively. After preheating in a water bath at 40 °C, the two solutions were thoroughly mixed, and the reaction was performed for 10 min. The reaction was terminated by adding 9 mL of 0.01 mol/L HCl, and the absorbance value was measured at 235 nm. The activity was (U·mL^−1^) expressed as one enzyme activity unit (U) per minute per milliliter of enzyme solution catalyzing the production of 1 g of galacturonic acid at 40 °C.

The PGTE and PMTE activities were determined following Yang et al.’s method [25] with minor modifications. The reaction mixture consisted of 1.0 mL enzyme solution, 1.0 mL of gly-NaOH buffer (50 mM, pH 9.0), 1.0 mL of substrate (10 g/L polygalacturonic acid), and 1.0 mL of 3 mM CaCl_2_. The mixture was reacted in a water bath at 30 °C for 10 min, and the absorbance value at 232 nm was measured. The activity (U·mL^−1^) was expressed as the substrate release of 1 μmol of unsaturated aldehyde acid as one unit per minute per mL of enzyme solution at 30 °C.

### 2.6. Measurements of Total Phenolic and Flavonoid Contents

Total phenolic and flavonoid acid contents were analyzed following Zhang et al.’s method [15]. Briefly, 3 g of frozen tissue was taken and mixed with 5 mL of pre-cooled 1 g L^−1^ HCL-methanol solution, fully ground and extracted under ice bath conditions, and the homogenate was centrifuged at 12,000× *g* and 4 °C for 10 min. The absorbance value of the supernatant was measured at 280 nm and 320 nm, respectively. The total phenolic content was expressed as OD_280_/g FW, and the flavonoid content was expressed as OD_325_/g FW.

### 2.7. Assays of the Activities of Key Enzymes Involved in Phenylpropane Metabolism

The activities of PAL, C4H, and 4CL were evaluated following the method of Li et al. [21] with some adjustments. Determination of PAL activity: 3 g of frozen tissue was added to 3 mL of 0.1 mol/L boric acid buffer, pH 8.8, fully ground under ice bath conditions, and then centrifuged at 10,000× *g*, 4 °C for 20 min to collect the supernatant. Then 0.5 mL crude enzyme solution was added with 0.5 mL of 0.02 mol/L phenylalanine boric acid buffer and 3 mL distilled water and mixed well. Then, the absorbance value was measured after the reaction at 30 °C water bath for 30 min. The blank was with phenylalanine boric acid buffer instead of enzyme solution. The change of absorbance per unit mass of the substance to be measured within an hour was 0.01 as 1 active unit (U). Determination of 4CL activity: 3 g of frozen tissue was added to 3 mL of Tris-HCL buffer at 4 °C, fully ground into homogenate in an ice bath, and the mortar was rinsed with 2 mL of extraction buffer. The homogenate combined twice was placed in a centrifuge tube and centrifuged at 10,000× *g*, 4 °C for 20 min. The supernatant was immediately used for the determination of 4CL activity. The change of absorbance per unit mass of the substance to be measured within an hour was 0.01 as 1 active unit (U). Determination of C4H activity: 3 g of frozen tissue was added to 3 mL Tris-HCl buffer at 4 °C and centrifuged at 10,000× *g*, 4 °C for 20 min. The reaction solution comprised 2.2 mL of 50 mmol/L Tris-HCl buffer (pH 8.9) and 0.8 mL of crude enzyme. Subsequently, the reaction solution was used to measure the absorbance at 340 nm. The change of absorbance per unit mass of the substance to be measured within an hour was 0.01 as 1 active unit (U) at 30 °C, U·g^−1^ FW.

### 2.8. Total RNA Extraction and Real-Time Quantitative PCR (RT-qPCR) Analysis

The extraction of total RNA from pulp tissue was performed following Wang et al. [26] with minor modifications. Briefly, total RNA was extracted by grinding 1 g of tissue into powder and mixed with 1.5 mL of 20 g L^−1^ acetyl trimethyl ammonium bromide (CTAB) solution. RNA purity and integrity were determined by agarose electrophoresis. First-strand cDNA was synthesized using the Fast Quant RT Kit (Tiangen, Beijing, China) and used for qRT-PCR. The primer sequences of the selected genes were designed and synthesized by Sangon Biotech (Shanghai, China). The qRT-PCR was conducted using LightCycle 96 (Roche, Switzerland). The analysis of the relative expression of all the genes followed the approach of Zhu et al. [27]. Gene expression levels were calculated using 2^−ΔΔt^ method, compared with the internal reference gene, *Pcactin*. Primer sequences of the selected genes and *Pcactin* are listed in Table 1,

### 2.9. Data Analyses

Results are expressed as mean ± SE. Data were analyzed by one-way ANOVA, followed by Duncan’s test using software SPSS 26.0 (SPSS, Inc., Chicago, IL, USA). *p* < 0.05 was recognized as a significant difference.

## 3. Results

### 3.1. Symptom on ‘Huangguan’ Pear Fruit Caused by R. solani 

**Figure 1:**
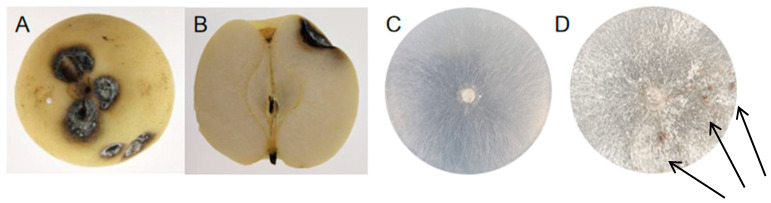
Symptoms of postharvest fruit rot caused by *R. solani* in ‘Huangguan’ pear. (**A**) External symptoms; (**B**) Internal symptoms; (**C**) Colony on PDA; (**D**) Sclerotinia morphology on PDA.

Symptoms began to appear 24 h after injury to the vaccination site, and visible lesions were formed after 48 h. The initial symptoms appeared on the surface of the pear fruit, manifested as dark brown spots. The spots gradually enlarged and eventually gathered together to form a large lesion, with the lesion color deepening to brown-black and the lesion depression forming a cavity. The boundary between diseased fruit pulp and healthy fruit pulp was not clear. A fruit may have several disease spots, and over time, the gray-white mycelium covers the entire surface of the fruit (Figure 1), eventually rotting.

### 3.2. Therapeutic and Protective Effects of Thifluzamide on Pear Fruit Rot 

**Figure 2:**
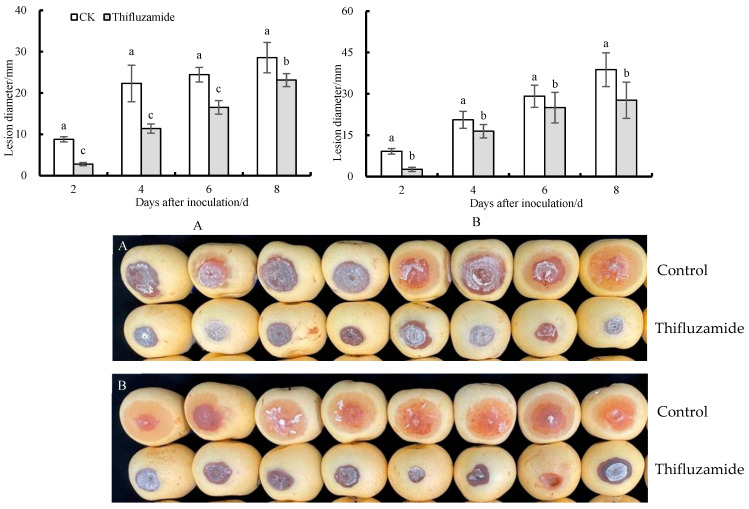
Effects of thifluzamide on lesion diameter of the fruit rot. (**A**) is the therapeutic effect, (**B**) is the protective effect. Bars indicate standard error (±SE). Letters above columns in the graphs denote significant differences at the level of *p* < 0.05.

As shown in Figure 2, thifluzamide treatment exhibited a strong therapeutic effect, and the average lesion expansion rate after treatment was significantly lower than the control group. The protective effect of thifluzamide was shown by decreasing the lesion diameter compared to the control.

### 3.3. Activities of CWDEs Secreted by R. solani

**Figure 3:**
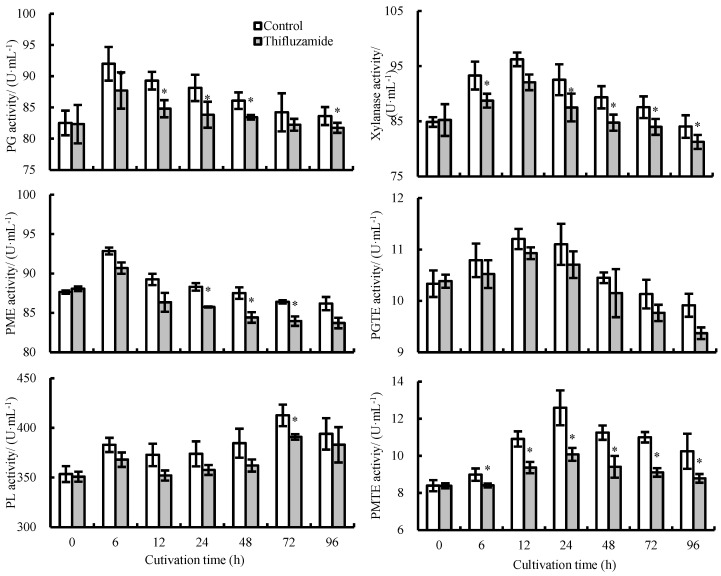
Effects of thifluzamide treatment on the activities of PG, PME, PL, xylanase, PGTE, and PMTE secreted by *R. solani.* Bars indicate standard error (±SE). Asterisks denote significant differences (*p* < 0.05).

The activities of CWDEs in the thifluzamide-treated *R. solani* were lower than those in the control during incubation (Figure 3). PG and PME activities increased dramatically from 0 to 6 h in the control and then decreased from 6 to 96 h, with the highest activities of PG and PME observed at 6 h. The PL activity of the control and treatment groups increased from 0 to 6 h, decreased from 6 to 12 h, increased from 12 to 72 h, and then decreased from 72 to 96 h, reaching its peak at 72 h. The activities of xylanase and PGTE showed a comparable trend during incubation. The activities increased from 0 to 12 h, reached their maximum activities at 12 h, and then decreased from 12 to 96 h. In the control group, the activity of PMTE increased from 0 to 24 h and decreased from 24 to 96 h. The activity of PMTE in the treatment group was significantly lower than that of the control group. At 24 h, the activity of PMTE in the control group was 1.25 times higher than that in the treatment group.

### 3.4. Effects of Thifluzamide Treatment on PAL, C4H, and 4CL Activities in Pears

**Figure 4:**
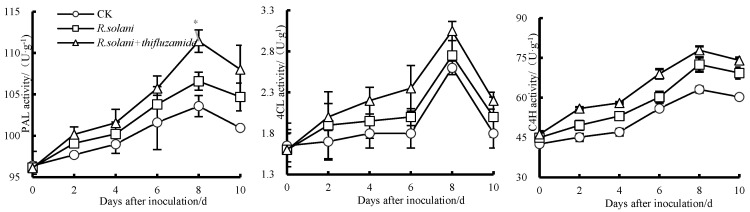
Effects of postharvest thifluzamide treatment on the activities of PAL, 4CLand C4H in *R. solani*-inoculated ‘Huangguan’ pears. Bars indicate standard error (±SE). Asterisks denote significant differences (*p* < 0.05).

As shown in Figure 4, PAL activity in the three groups increased from day 0 to 8 before decreasing from day 8 to 10. Compared to the control group, the PAL activity in the *R. solani*-inoculated group increased from day 0 to 10, and the PAL activity of the *R. solani* + thifluzamide group increased, compared to the *R. solani* group, reaching an apex at day 8. C4H activity amongst the three groups also increased from day 0 to 8 and decreased from day 8 to 10. Similarly, the activity of C4H in the *R. solani* + thifluzamide group increased from day 0 to 10. The highest C4H activity was observed on the 8th day. Additionally, 4CL activity increased from day 0 to 8 and then decreased from day 8 to 10 in all three groups.

### 3.5. Effects of Thifluzamide Treatment on the Expression of PcPAL, PcC4H, Pc4CL, Pcβ-1,3-GA, PcLCH, PcF3H, and PcDFR in Pears

**Figure 5:**
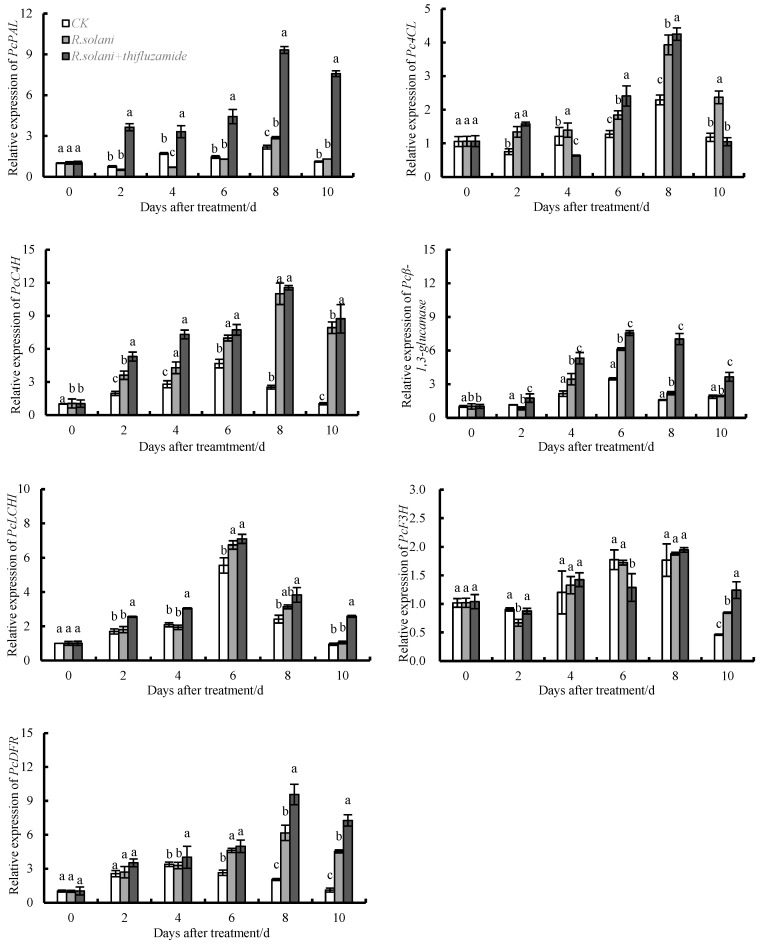
Effects of thifluzamide treatment on the expression of *PcPAL*, *PcC4H*, *Pc4CL*, *Pcβ-1,3-GA*, *PcCHI*, *PcF3H*, and *PcDFR* in *R. solani-inoculated* ‘Huangguan’ pears. Bars indicate standard error (±SE). Letters above columns in the graphs denote significant differences at the level of *p* < 0.05.

As shown in Figure 5. The expression of *PcPAL* peaked at day 8. Compared to the control group, *R. solani* inoculation up-regulated the expression of *PcPAL* on days 8 and 10, and the expression levels of *PcPAL* in the *R. solani* + thifluzamide group were higher than those in the *R. solani* group on days 2–10. The expression of *PcPAL* increased 1.32 times and 1.17 times after *R. solani* inoculation for 8 and 10 d, respectively. The expression levels of *PcPAL* in the *R. solani* + thifluzamide group were 7.13-, 4.71-, 3.41-, 3.24-, and 5.80 times higher than those in the *R. solani* group on days 2, 4, 6, 8, and 10, respectively. Compared to the control group, the expression of *PcC4H* in the *R. solani* group increased and reached its peak at day 8. On days 2, 8, and 10, the expression levels of *PcC4H* in the *R. solani* group were significantly higher than those in the control group (1.84, 4.35, and 7.74 times, respectively). Compared to the *R. solani* group, the expression of *PcC4H* in the *R. solani* + thifluzamide group was up-regulated and reached its peak at day 8. On days 2, 4, and 10, the expression levels of *PcC4H* in the *R. solani* + thifluzamide group were significantly higher than those in the *R. solani* group (1.47, 1.05, and 1.10 times, respectively). *Pc4CL* expression also peaked at day 8. Compared to the control, the expression levels of *Pc4CL* were increased by 1.78, 1.15, 1.45, 1.71, and 2.01 times by *R. solani* inoculation on days 2, 4, 6, 8, and 10, respectively. Compared to the *R. solani* group, the expression levels of *Pc4CL* in the *R. solani* + thifluzamide group were 1.57, 2.41, and 4.25 times higher than those in the *R. solani* group on days 2, 6, and 8, respectively. In addition, *R. solani* inoculation up-regulated the expression of *Pcβ-1,3-GA* on days 4, 6, 8, and 10, with 1.60, 1.76, 1.38, and 1.03 times higher than those in the control group, respectively. *R. solani* + thifluzamide up-regulated the expression of *Pcβ-1,3-GA* on days 2, 4, 6, 8, and 10, with 2.09, 1.54, 1.23, 3.19, and 1.87 times higher than those in the *R. solani* group, respectively. The expression of *PcCHI* in three groups increased from day 0 to 6 and then decreased from day 6 to 10. Compared to the control group, the *R. solani* inoculation up-regulated the expression of *PcCHI* on days 2 and 6–10. Compared to the *R. solani* group, the expression of *PcCHI* in the *R. solani* + thifluzamide group increased from day 2 to 10. The expression level of *PcCHI* reached the highest level on day 6, and the *R. solani* group was 1.21 times higher than that of the control group, and the *R. solani* + thifluzamide group was 1.05 times than that of the *R. solani* group. *PcF3H* expression in the three groups increased from day 0 to 8 and then decreased from day 8 to 10. On days 2 and 10, the expression of *PcF3H* in the *R. solani* group was higher than those in the control group, and on days 2, 4, and 8–10, the expression levels of *PcF3H* in the *R. solani* + thifluzamide group were higher than those in the *R. solani* group. *PcF3H* expression reached the highest level at day 8. The expression levels of *PcDFR* in all three groups were gradually increased.

### 3.6. Total Phenolic and Flavonoid Contents

**Figure 6:**
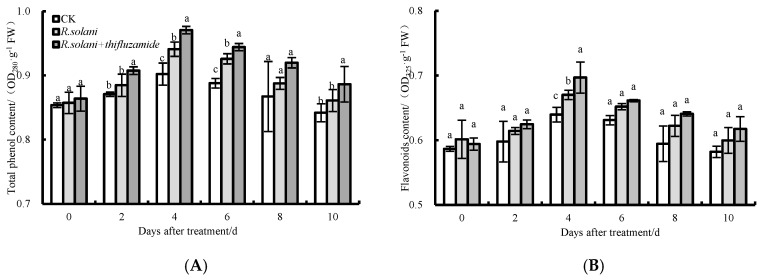
Total phenolic (**A**) and flavonoid (**B**) contents in pear fruit after thifluzamide treatment during storage at 20 ± 1 °C with 75 ± 5% RH. Bars indicate standard error (±SE). Letters above columns in the graphs denote significant differences at the level of *p* < 0.05.

The relative contents of total phenolic and flavonoid in the three groups increased from 0–4 d after storage; then, the levels tended to decrease (Figure 6). From 0 to 10 days, the contents of total phenolic and flavonoid in the *R. solani* + thifluzamide group were higher than those in the *R. solani* group. The total phenolic contents in the *R. solani* + thifluzamide group were significantly higher than those in the *R. solani* group on days 2, 4, and 6, while the flavonoid content was significantly higher than that in the *R. solani* group on day 6. 

## 4. Discussion

In the process of invading the host, pathogenic fungi secrete CWDEs, such as cellulase, pectinase, and protease, to digest the structure of plant cell walls [28]. Cellulase, comprised primarily of Cx and β-Glu, continuously hydrolyzes the natural cellulose in the cell wall into small glucose molecules. Xylanase is an enzyme that hydrolyzes the polysaccharides that form the cell walls of plants. Pectinase, a kind of multi-component complex enzyme, can degrade the pectin in the plant body, dissociate and rot soft tissue, and promote the senescence or death of the plant. In this study, the activities of PG, PME, PGTE, PMTE, Cx, β-Glu, xylanase, and PL secreted by *R. solani* were inhibited by thifluzamide treatment with the prolongation of culture time. PG may promote the ability of other CWDEs to attack substrates. The current study demonstrated that thifluzamide resisted the invasion of *R. solani* in pears. Among the numerous CWDEs produced by *R. solani*, pectinase is produced and secreted in large quantities to help mycelium invade the host. This enzyme works with cellulase to participate in disease expansion [29]. This is inconsistent with the results of this study. The discrepancy may be related to the host infection mode, host type, hyphal infection period, or the infection processes of different fusion groups of *R. solani*. Cellulose, as the skeleton of the cell wall, is usually composed of white crystalline microfilaments. Pectin is a polysaccharide component of the cell wall, which plays an important role in controlling porosity, cell adhesion, cell expansion, and bacterial resistance [30,31]. 

Phenylpropane metabolism is an important secondary metabolic pathway. PAL is the rate-limiting enzyme in this pathway that regulates the biosynthesis of phenols and lignin [32]. C4H and 4CL are the two key enzymes involved in the synthesis of phenolic compounds [18]. Phenolic compounds play an important role in disease resistance due to their antibacterial, antioxidant, and free radical-eliminating properties [33]. Phenolic compounds can even be converted into toxic quinones to act directly on pathogens and reduce the occurrence of diseases [15]. In this experiment, the activity of PAL in the control group increased first and then decreased, and the PAL activity of pear fruits was increased both by *R. solani* alone and *R. solani* in conjunction with thifluzamide treatment. Thifluzamide treatment displayed the strongest capability to induce PAL activity in the fruits. It was reported that sodium phosphate treatment increased PAL activity in apple fruits during storage and improved their resistance to black spot disease [34] (Ge et al., 2019). 4CL is one of the key enzymes in the downstream branches of phenylpropane metabolism, catalyzing the conversion of ferulic acid, p-coumaric acid, cinnamic acid, and caffeic acid to coumaroyl-CoA [35]. In this study, it was found that 4CL activity in the pear fruits treated with thifluzamide remained at higher levels when compared to the group only inoculated with *R. solani*. A study in Dongzao fruits found that exogenous caffeic acid and epicatechin treatment activated 4CL activity and promoted the accumulation of downstream products, thus improving fruit resistance [31]. The changing trend of CWDE activity in pear fruits treated with thifluzamide was basically the same as that of *R. solani*-treated fruits, but the activity was lower than that of *R. solani* inoculated only during the entire storage period. These results indicated that thifluzamide treatment could not directly increase the activities of disease-resistance-related enzymes when the fruit was not seriously infected by pathogens but could strongly activate these defense responses when the fruit was infected by pathogen activators. The results showed that thifluzamide treatment increased the contents of total phenolic and flavonoid in the pear fruits, demonstrating a uni-modal trend. It was also found that sodium phosphate treatment promoted the accumulation of total phenols and flavonoids during storage [18]. These results indicated that thifluzamide promoted the accumulation of disease-resistant substances by activating phenylpropane metabolism in the pear fruits.

This study also found that thifluzamide treatment significantly increased the expression of the key genes, *PcPAL*, *PcC4H*, *Pc4CL*, *Pcβ-1,3-GA*, *PcLCH*, *PcF3H* and *PcDFR* involved in phenylpropane metabolism. After inoculation with *R. solani*, the expression of the CWDEs genes was up-regulated, and the expression level was higher than that without inoculation and thifluzamide treatment, indicating that the thifluzamide treatment could well protect the pear fruits from the infection of *R. solani*. However, the expression of the genes related to disease resistance was up-regulated in the treated pear fruits, and the expression level was higher than those of the untreated and the *R. solani* treated fruits, indicating that the treated pear fruits may have stronger disease resistance. It was also reported that the expression levels of phenylpropane metabolism-related genes in the pear fruits treated with γ-aminobutyric acid were increased, which enhanced the resistance of the fruit to *Penicillium expansum* [36]. Salicylic acid, nitride oxide, and UV-C treatment of citrus, peach, and tomato fruits also significantly up-regulated the expression levels of *PAL*, *C4H*, *4CL,* and *CHI*. This accelerated the production rate of downstream secondary metabolites and thus reduced fruit morbidity [37,38]. These results indicated that thifluzamide treatment affected the activities of CWDEs in ‘Huangguan’ pears by regulating their expression, thus improving their storage quality. At the same time, thifluzamide increased the expression of the key genes involved in the phenylpropane metabolism pathway in pear fruits. This promoted the accumulation of the resistance substances, including total phenolic and flavonoid, and improved its disease resistance.

## 5. Conclusions

Thifluzamide was found to exhibit a protective effect by inhibiting the activities of CWDEs secreted by *R. solani*. It also had a therapeutic effect by enhancing the ability of the phenylpropanoid pathway in pears. Therefore, thifluzamide can be used as an effective pre-harvest prevention and control fungicide for pear fruit rot caused by *R. solani*.

## Figures and Tables

**Table 1:** Primer sequences of the selected genes used in this study.

Gene	Primer Sequence	NCBI Number
*PcPAL*	F: CGTATGGTGGCGGAGTACAGAAAG	NM_001319807.1
	R: GCTATTGCTGCAACTTGGGAAATGG	
*PcC4H*	F: GGCAGTTCACTCTCCCACACAAC	KF663548.1
	R: TTTCCAGGAGGAGGAGGTCCATTG	
*Pc4CL*	F: CCTCTTCCCTCAAGCACCAATTCAG	XM_009372686.2
	R: ATGGGGATGTCGGGGAGTTTGG	
*Pcβ-1,3-GA*	F: CTTGATGCCAGCCCTGCAAATAAC	JX127223.1
	R: GCCCGATGCCATTGCTTTTGTAC	
*PcLCH*	F: AATGGCTTCTGCTTTTCTCAATGGC	KP202179.1
	R: TCACTATCTTCACCAACGGCTTCAC	
*PcF3H*	F: TGGCTCCTGCTACTACGCTCAC	KC460396.1
	R: AACCTTTGGACGCTCGTCTTCG	
*PcDFR*	F: CGAAACACCCAACCGTTTAGTTCAG	MF489221.1
	R: TTGTCCTTGCTCTTCAGTGCTCAC	
*Pcactin*	F: AGAGCATCCAGTCCTCCTGACAG	AF386514.1
	R: GCCTGAATTGCAACGTACATAGCC	

## Data Availability

The data that has been used is confidential.

## References

[1] Joyce (2022). Garden. https://www.garden.eco/where-do-pears-grow.

[2] Warmly J. (2022). Northern Nester. https://northernnester.com/types-of-pears.

[3] Tomasz C., Jan O., Ireneusz K., Sabina L. (2019). Effect of abiotic stress factors on polyphenolic content in the skin and flesh of pear by UPLC-PDA-Q/TOF-MS. Eur. Food Res. Technol..

[4] Wang H., Wang Z., Zhang M., Jia B., Heng W., Ye Z., Zhu L., Xu X. (2018). Transcriptome sequencing analysis of two different genotypes of Asian pear reveals potential drought stress genes. Tree Genet. Genomes.

[5] Kou X.H., Li Y.F., Zhang Y., Jiang B.L., Xue Z.H. (2018). Gene expression and activity of enzymes involved in sugar metabolism and accumulation during “Huangguan” and “Yali” pear fruit development. Trans. Tianjin Univ..

[6] Ma Y.R., Yang M.G., Wang J.J., Jiang C.Z., Wang Q.G. (2016). Application of Exogenous Ethylene Inhibits Postharvest Peel Browning of ‘Huangguan’ Pear. Front. Plant Sci..

[7] Jia X.H., Xu J.N., Wu Y.S., Zhang X.N., Du Y.M., Wang W.H. (2022). Isolation, identification and artificial inoculation of *Rhizoctonia solani* on pear during storage. Hortic. Plant J..

[8] Ning X.X., Su Y., Ma Y., Ning X.Y., Zhang J.H. (2019). Advances in research on *Rhizoctonia solani*. Heilongjiang Agric. Sci..

[9] Zhang X.Y., Huo H.L., Xi X.M., Liu L.L., Yu Z., Hao J.J. (2016). Histological observation of potato in response to *Rhizoctonia solani* infection. Eur. J. Plant Pathol..

[10] Prashantha S.T., Bashyal B.M., Krishnan S.G., Dubey H., Solanke A.U., Prakash G., Aggarwalet R. (2021). Identification and expression analysis of pathogenicity-related genes of *Rhizoctonia solani* anastomosis groups infecting rice. 3 Biotech.

[11] Joomdok J., Saepaisan S., Sunpapao A., Pongpisutta R., Monkham T., Sanitchon J., Chankaew S. (2021). Identification of *Rhizoctonia solani*, as the cause of rice sheath blight and the source of its resistance, from Thai indigenous lowland rice germplasm. Euphytica.

[12] Brito N., Espino J.J., González C. (2006). The endo-beta-1,4-xylanase xyn11A is required for virulence in *Botrytis cinerea*. Mol. Plant-Microbe Interact..

[13] Siah A., Deweer C., Duymec F., Sanssené J., Durand R., Halama P., Reignault P. (2010). Correlation of in planta endo-beta-1,4-xylanase activity with the necrotrophic phase of the hemibiotrophic fungus *Mycosphaerella graminicola*. Plant Pathol..

[14] Ge Y.H., Wei M.L., Li C.Y., Chen Y.R., Lv J.Y., Meng K., Wang W.H., Li J.R. (2018). Reactive oxygen species metabolism and phenylpropanoid pathway are involved in disease resistance against *Penicillium expansum* in apple fruit induced by ϵ-poly-l-lysine. J. Sci. Food Agric..

[15] Zhang M.Y., Wang D.J., Gao X.X., Yue Z.Y., Zhou H.L. (2020). Exogenous caffeic acid and epicatechin enhance resistance against *Botrytis cinerea* through activation of the phenylpropanoid pathway in apples. Sci. Hortic..

[16] Ge Y.H., Dong X., Wei M.L., Li C.Y., Jiang C.N., Chen Y.R. (2018). Effect of chitosan combined with polylysine on blue mould of apple fruit and antioxidant enzymes and phenylpropanoid pathway. Sci. Technol. Food Ind..

[17] Gacnik S., Veberic R., Marinovic S., Halbwirth H., Mikulic-Petkovsek M. (2021). Effect of pre-harvest treatments with salicylic and methyl salicylic acid on the chemical profile and activity of some phenylpropanoid pathway related enzymes in apple leaves. Sci. Hortic..

[18] Ge Y.H., Tang Q., Li C.Y., Duan B., Li X., Wei M.L., Li J.R. (2019). Acibenzolar-S-methyl treatment enhances antioxidant ability and phenylpropanoid pathway of blueberries during low temperature storage. LWT-Food Sci. Technol..

[19] Deng Y.X., Lu S.F. (2017). Biosynthesis and regulation of phenylpropanoids in plants. Crit. Rev. Plant Sci..

[20] Jiang H., Wang B., Ma L., Zheng X.Y., Gong D., Xue H.L., Bi Y., Wang Y., Zhang Z., Prusky D. (2019). Benzo-(1, 2, 3)-thiadiazole-7-carbothioic acid s-methyl ester (BTH) promotes tuber wound healing of potato by elevation of phenylpropanoid metabolism. Postharvest Biol. Technol..

[21] Li L., Liu Q.L., Xue H.L., Bi Y., Raza H., Zhang R., Carelle J.K., Peng H., Long H.T., Prusky D. (2022). Acetylsalicylic acid (ASA) suppressed Fusarium rot development and neosolaniol (NEO) accumulation by activating phenylpropane metabolism in muskmelon fruit. Eur. J. Plant Pathol..

[22] Jia L.L., Li Y., Liu G.S., He J.G. (2022). Acidic electrolyzed water improves the postharvest quality of jujube fruit by regulating antioxidant activity and cell wall metabolism. Sci. Hortic..

[23] Yuan H.B., Yuan M.J., Shi B.K., Wang Z.N., Huang T.X., Qin G.H., Hou H., Wang L., Tu H.T. (2022). Biocontrol activity and action mechanism of *Paenibacillus polymyxa* strain Nl4 against pear Valsa canker caused by *Valsa pyri*. Front. Microbiol..

[24] Ahn S.E., Wang M.H., Lee A.Y., Hwang Y.S. (2014). Effects of short-term treatment of high-pressure CO_2_ on the changes in fruit quality during the storage of ‘Maehyang’ strawberries. Korean J. Agric. Sci..

[25] Yang Y.Q., Lan B., Jian Y.L., Chang D.D., Zhang S.L., Li X.M. (2015). Infection process and pathogenic mechanism of *Phomopsis asparagi*, the Asparagus stem blight pathogen. Phytoparasitica.

[26] Wang Z., Jia C., Li J., Huang S., Xu B., Jin Z. (2015). Activation of salicylic acid metabolism and signal transduction can enhance resistance to Fusarium wilt in banana (*Musa acuminata* L. AAA group, cv. Cavendish). Funct. Integr. Genom..

[27] Zhu J., Li C.Y., Fan Y.T., Qu L.H., Huang R., Liu J.X., Zhang C.Y., Ge Y.H. (2022). γ-Aminobutyric acid regulates mitochondrial energy metabolism and organic acids metabolism in apples during postharvest ripening. Postharvest Biol. Technol..

[28] Joseph A.K. (2020). A Study on the Biocontrol Activity of *Rhodotorula mucilaginosa* Combined with Salicylic Acid Against *Penicillium digitatum* Infection in Oranges.

[29] Chen M.Y., Lin H.T., Hung Y.C., Zhang S., Lin Y.F., Chen Y.H. (2015). Regulation of 2,4-dinitrophenol and adenosine triphosphate on disease development, energy status, and respiratory metabolism of *Phomopsis longanae* chi-infected longan fruit. Mod. Food Sci. Technol..

[30] Lampugnani E.R., Khan G.A., Somssich M., Persson S. (2018). Building a plant cell wall at a glance. J. Cell Sci..

[31] Bellincampi D., Cervone F., Lionetti V. (2014). Plant cell wall dynamics and wall-related susceptibility in plant-pathogen interactions. Front. Plant Sci..

[32] Zhang J.R., Zhang L.L., Chang L.L., Zhang S.Y. (2020). Effect of chitosan combined with sodium silicate on phenylpropanoid metabolism and pathogenesis-related proteins of postharvest winter jujube. Sci. Technol. Food Ind..

[33] Wang Y., Ji D.C., Chen T., Li B.Q., Zhang Z.Q., Qin G.Z., Tian S.P. (2019). Production, signaling, and scavenging mechanisms of reactive oxygen species in fruit-pathogen interactions. Int. J. Mol. Sci..

[34] Ge Y.H., Chen Y.R., Li C.Y., Wei M.L., Li X., Tang Q., Duan B. (2019). Effect of trisodium phosphate treatment on black spot of apple fruit and the roles of anti-oxidative enzymes. Physiol. Mol. Plant Pathol..

[35] Ge Y.H., Duan B., Li C.Y., Tang Q., Li X., Wei M.L., Chen Y.R., Li J.R. (2018). γ-Aminobutyric acid delays senescence of blueberry fruit by regulation of reactive oxygen species metabolism and phenylpropanoid pathway. Sci. Hortic..

[36] Yu C., Zeng L.Z., Sheng K., Chen F.X., Zhou T., Zheng X.D., Yu T. (2014). γ-Aminobutyric acid induces resistance against *Penicillium expansum* by priming of defence responses in pear fruit. Food Chem..

[37] Zhou Y.H., Ma J.H., Xie J., Deng L.L., Yao S.X., Zeng K.F. (2018). Transcriptomic and biochemical analysis of highlighted induction of phenylpropanoid pathway metabolism of citrus fruit in response to salicylic acid, *Pichia membranaefaciens* and oligochitosan. Postharvest Biol. Technol..

[38] Liu C.H., Zheng H.H., Sheng K.L., Liu W., Zheng L. (2018). Effects of postharvest UV-C irradiation on phenolic acids, flavonoids, and key phenylpropanoid pathway genes in tomato fruit. Sci. Hortic..

